# Sensitivity of Shipborne GNSS Estimates to Processing Modeling Based on Simulated Dataset

**DOI:** 10.3390/s23146605

**Published:** 2023-07-22

**Authors:** Aurélie Panetier, Pierre Bosser, Ali Khenchaf

**Affiliations:** 1 PIM UMR 6285 CNRS, Lab-STICC (Laboratoire des sciences et techniques de l’information, de la communication et de la connaissance), ENSTA Bretagne, 29200 Brest, France; aurelie.panetier@ensta-bretagne.org; 2 M3 UMR 6285 CNRS, Lab-STICC (Laboratoire des sciences et techniques de l’information, de la communication et de la connaissance), ENSTA Bretagne, 29200 Brest, France; pierre.bosser@ensta-bretagne.fr

**Keywords:** shipborne GNSS, PPP, ZWD, simulation, processing modeling, Kalman filter

## Abstract

The atmospheric water vapor is commonly monitored from ground Global Navigation Satellite System (GNSS) measurements, by retrieving the tropospheric delay under the Zenith Wet Delay (ZWD) component, linked to the water vapor content in the atmosphere. In recent years, the GNSS ZWD retrieval has been performed on shipborne antennas to gather more atmospheric data above the oceans for climatology and meteorology study purposes. However, when analyzing GNSS data acquired by a moving antenna, it is more complex to decorrelate the height of the antenna and the ZWD during the Precise Point Positioning (PPP) processing. Therefore, the observation modeling and processing parametrization must be tuned. This study addresses the impact of modeling on the estimation of height and ZWD from the simulation of shipborne GNSS measurements. The GNSS simulation is based on an authors-designed simulator presented in this article. We tested different processing models (elevation cut-off angle, elevation weighting function, and random walk of ZWD) and simulation configurations (the constellations used, the sampling of measurements, the location of the antenna, etc.). According to our results, we recommend processing shipborne GNSS measurements with 3° of cut-off angle, elevation weighting function square root of sine, and an average of 5 mm·h${}^{-1/2}$ of random walk on ZWD, the latter being specifically adapted to mid-latitudes but which could be extended to other areas. This processing modeling will be applied in further studies to monitor the distribution of water vapor above the oceans from systematic analysis of shipborne GNSS measurements.

## 1. Introduction

The Global Navigation Satellite System (GNSS) measurement is based on an electromagnetic signal emitted in the L-band by GNSS satellites around the Earth and received by GNSS antennas at the Earth’s surface [1]. From the measurements between a receiving antenna and each satellite, we can compute the antenna coordinates using the pseudorange or carrier phase observable of the signal. Precise point positioning (PPP) processing is an absolute positioning method that uses the position of the observed satellites, the pseudorange, and the carrier phase difference between the satellites and the signal received from the antenna [2,3].

Different aspects must be taken into account to obtain an accurate PPP positioning. First, the receiver and satellite clocks must be well-synchronized to obtain precise positioning, but they are subject to some errors. Contrary to the satellite clock continuously monitored by ground reference stations, the receiver clock errors are unknown, so they have to be estimated. On the other hand, the GNSS signals are disturbed by the medium through which they pass: the ionosphere at higher altitudes [4], and the troposphere at lower altitudes [5]. The ionosphere influences different frequencies in different ways. As the GNSS signal is composed of at least two frequencies, it is possible to remove the first-order effect of the ionosphere on the GNSS signal by using a linear combination of both frequencies [2,6]; a second-order correction could also be applied for a better correction of this effect [7]. The impact of the troposphere on the GNSS signal causes a delay whose zenith component is called the Zenith Troposphere Delay (ZTD) [8]. This delay is due to the refractivity of the medium whose density varies when the signal propagates toward the Earth’s surface. The change in density modifies both the path of propagation of the signal and its velocity [9]. Then the propagation time is longer than without the troposphere. The main component of ZTD is the Zenith Hydrostatic Delay (ZHD), which depends only on the atmospheric pressure at the height of the antenna [10,11]. The ZHD component can be estimated from empirical models or meteorological surface measurements and set up as an a priori component in the modeling part before calculating the other PPP variables [12,13,14]. Indeed, the a priori value of ZHD can be correctly computed thanks to a simple pressure, temperature, and humidity sensor located near the GNSS antenna, or by using a reanalysis such as ERA5 or a model such as VMF3 [11,15]. These models give suitable pressure results at sea as we are near the surface. The other component of the ZTD depends on the water vapor in the atmosphere, which is highly variable in space and time.This wet part, called Zenith Wet Delay (ZWD), cannot be corrected a priori for the GNSS analysis [8,9]. As a result, the clock error and the ZWD are estimated with position [16], resulting in a positioning error of less than 1 cm [3,17].

Several studies focused on the precise extraction of ZWD from raw GNSS measurements [18,19,20,21,22,23]. ZWD has long been estimated from ground GNSS antennas [20,24,25,26,27]. It is currently assimilated into numerical weather prediction models [28], providing a dense data set in some areas such as Europe [29], Australia [30], and North America [31]. Weather prediction and climate studies are made easier and more precise on land since the GNSS provides an all-weather and high temporal resolution with very good accuracy at a lower cost [32,33,34].

The vast majority of studies on GNSS for atmospheric water vapor measurements are based on fixed ground reference stations [24,35]. These measurements allow for spatial and temporal monitoring of atmospheric water vapor over land. However, contrary to land, data collected over oceans are sparse, mainly coming from satellite-mounted radiometers or radiosondes launched from islands or shipborne platforms, providing low space and time resolution [36,37]. However, the most violent weather phenomena originate at sea [38,39,40]. It is then important to obtain more data on the sea to study these phenomena. This is the reason why more and more studies are retrieving water vapor at sea by processing shipborne GNSS antennas [22,23,27,41,42,43,44].

GNSS PPP processing results are sensitive to the processing modeling [45], mainly due to the stochastic nature of the parameters and their intercorrelation. Some studies have already been carried out on the effects of processing modeling. Hadas et al. [46] proposed a new weighting function on satellite elevation and compared it with the processing weighting functions usually applied. Bar-Sever et al. [47] recommended processing random walk values, and Selle and Desai [48] showed that random walk processing is weighting function dependent. Kačmařík et al. [49] studied the impact of processing modeling on tropospheric gradient estimation, recommending using 3° of cut-off angle.

Processing GNSS data with appropriate modeling is especially relevant at sea. Indeed, the carrying vessel is moving, so many parameters have to be estimated simultaneously with low constraints. A previous study has been carried out to investigate the impact of modeling on the estimation of height and ZWD using GNSS measurements from a shipborne antenna [50]. In this previous study, GNSS raw data acquired from the antenna mounted on a research vessel were processed for one month in PPP mode using Gipsy-Oasis II v6.4 (Jet Propulsion Laboratory (JPL), NASA, Pasadena, CA, USA). Comparisons of the height estimate with the sea surface height from the tide gauge of Brest Harbor and the ZWD estimate from the nearest ground GNSS reference antenna pointed out a modeling that may considerably improve the agreement with the reference data set. It seems therefore important to assess a set of modeling parameters for kinematic PPP processing of shipborne GNSS antennas, to make sure we achieve the best estimation possible of position and tropospheric delay when processing any shipborne antenna. As the studied vessel remained in Brest harbor, the acquisition conditions were favorable, which is not the case for an operation in the open sea [51]. The sea state is more diverse, and there are little data available to assess the results. Then, it seems necessary to generalize the processing modeling to a broader range of shipborne GNSS datasets.

This study aims at recommending a processing model for the systematic analysis of GNSS raw data acquired by shipborne antennas. Indeed, hundreds of research vessels (R/V) carrying a GNSS antenna travel the oceans across the World, acquiring GNSS raw data. The potential of these data is significant if their systematic analysis is possible. In order to test a series of processing modeling to a more typical dataset, we simulated a GNSS signal that would correspond to the signal acquired by a GNSS antenna as those mounted on R/V. We estimated the GNSS variables from this signal through a Kalman filter with different processing parameters. We compared the estimated height and ZWD to the simulated quantities. The realization of several simulations made it possible to determine statistically whether a configuration should be preferred. Using simulations also enables the data processing as many times as needed in a short period to test all the configurations altogether, as changing a processing parameter could affect the behavior of others [48]. Therefore, Section 2 explains how the simulation of the shipborne GNSS signals and their processing works and the different features. Section 3 presents and discusses the results in the studied processing configurations. It highlights a recommended modeling of the future systematic analysis processing of GNSS data acquired from R/V. Section 4 draws the conclusion and perspectives.

## 2. Method

### 2.1. Simulation of GNSS Measurements

#### 2.1.1. Carrier Phase Measurement Simulation

The simulated acquisition is made up by adding different simulated components, as explained in the following sections, such as in Equation (1).

(1) $$\phi_{IF_{i}}=C+H\cdot \sin\left(\epsilon \right)+\frac{ZWD}{\sin\left(\epsilon \right)}+PCV_{IF}\left(\alpha ,\epsilon \right)+MP_{IF}\left(\alpha ,\epsilon \right)+\eta_{IF}$$

where $\phi_{IF_{i}}$ denotes the linear ionosphere-free (IF, Section 2.1.4) combination simulated carrier phase from the i-th satellite, *C* denotes the receiver clock error, *H* denotes the simulated height, ZWD denotes the simulated troposphere wet delay, $PCV_{IF}\left(\alpha ,\epsilon \right)$ denotes the linear IF combination simulated antenna phase center variation, $MP_{IF}\left(\alpha ,\epsilon \right)$ denotes the simulated multipath, $\eta_{IF}$ denotes the simulated measurement noise, and α and ϵ denote the azimuth and elevation of the *i*-th satellite, respectively.

Equation (1) was inspired by Elosegui et al. [52] that modeled a ground-based antenna GPS signal with constant ambiguity, tropospheric zenith propagation delay, vertical coordinate variation, and multipath. However, King et al. [53] assumed fixed ambiguities by removing them from its simulation model. Then we decided to keep only the height variation, essential for our kinematic study, and the tropospheric variation from the Elosegui et al. simulation model, and we added the receiver clock error *C*, resulting in the first three terms of the simulated carrier phase of Equation (1). The three last terms in Equation (1) represent different measurement errors. All terms are explained in the following sections.

We used real 24-h broadcast satellite orbits at a 30-s time resolution from final products broadcasted by the International GNSS Station (IGS). Other real broadcast orbits have been tested, which show no significant impact on the simulation. Thus, as for the King et al. [53] simulation, satellite orbits and clocks are assumed to be known and free of errors. It is important to note that clock and orbit errors can be non negligible source of error in GNSS processing [54], but this point will not be discussed here. The azimuth α and elevation ϵ of the observed satellite in Equation (1) come from their ephemerides.

#### 2.1.2. Receiver Clock Error Simulation

Although it is possible to implement a clock error in this simulator through the *C* term, it has been set to zero throughout the day for this study, reproducing the choice of Elosegui et al. [52] to not simulate clock errors.

#### 2.1.3. Antenna Movements Simulation

A change in antenna height results in an additional signal path stretch expressed as H·sinϵ [55]. The simulated antenna height *H* integrates the simulated tide and heave to the mean sea level (MSL), as well as a constant height positioning $H_{0}=30$ m above sea surface, corresponding to an R/V antenna height.

The angular motions of the boat are neglected in this study. It could be interesting to study the impact of such movements on GNSS signal PPP processing because these movements can be very heavy on boats, especially during rough sea states.

The horizontal displacement of the antenna carried out by an R/V is not studied here, so its effect is not simulated for simplification purposes.

#### 2.1.4. Atmospheric Propagation Simulation

Our simulated GNSS carrier phase is based on a linear IF combination of two frequencies from each GNSS constellation, such as Equation (2).

(2) $$X_{IF}=IF1\cdot X_{1}-IF2\cdot X_{2}$$

where:

$$IF1=\frac{f_{1}^{2}}{f_{1}^{2}-f_{2}^{2}}$$

$$IF2=\frac{f_{2}^{2}}{f_{1}^{2}-f_{2}^{2}}$$

where $X_{IF}$ denotes the linear IF combination of the simulated variable, $f_{1}$ and $f_{2}$ the two frequencies for a given GNSS constellation, and $X_{1}$ and $X_{2}$ the corresponding simulated variable at each signal frequency.

The available frequencies depend on the constellation, as in Table 1.

The frequency we chose to select in Table 1 to simulate GLONASS signals corresponds to the lowest frequencies of the L1 and L2 bands. Indeed, the GLONASS system uses channels of frequencies separated by about 0.5 MHz to distinguish the signals coming from the different satellites of the constellation [56].

Using the linear combination of Equation (2), it is assumed that the ionospheric effect on GNSS signal measurement has been removed, so that the simulated ionosphere term does not appear in Equation (1). Thus, we call it the linear IF combination.

Because the troposphere hydrostatic delay is usually computed aside the estimation of the parameters, as explained in Section 1, we chose not to simulate it and we likened the total tropospheric delay to the ZWD in our study.

The measurement of the troposphere wet delay at the zenith of the antenna is simulated by setting a first ZWD value of about 0.15 m, and a random walk variability depending on the latitude of the study.

The water vapor delay at the zenith of the antenna is mapped to the GNSS signal path between a satellite at elevation ϵ and the antenna using mapping functions that involve a continuing fraction of sinϵ, such as GPT3 or VMF3 [57]. These mapping functions involve coefficients that can be fitted to radiosondes such as Niell Mapping Functions (NMF) [58], empirically based on numerical weather data such as Global Mapping Function (GMF) and GPT2 [59] recently updated to GPT3 [57], or derived from ray-tracing through actual data such as Vienna Mapping Functions (VMF1 and its refinement VMF3) [57,60]. All these mapping functions can be approximated to a simple $\frac{1}{\sin\epsilon }$, equivalent to suppose a flat earth, which still works well [55]. This is the simplified modeling we adopted for the troposphere wet delay on the signal path in our simulation.

#### 2.1.5. Antenna Phase Center Variation Simulation

**Figure 1:**
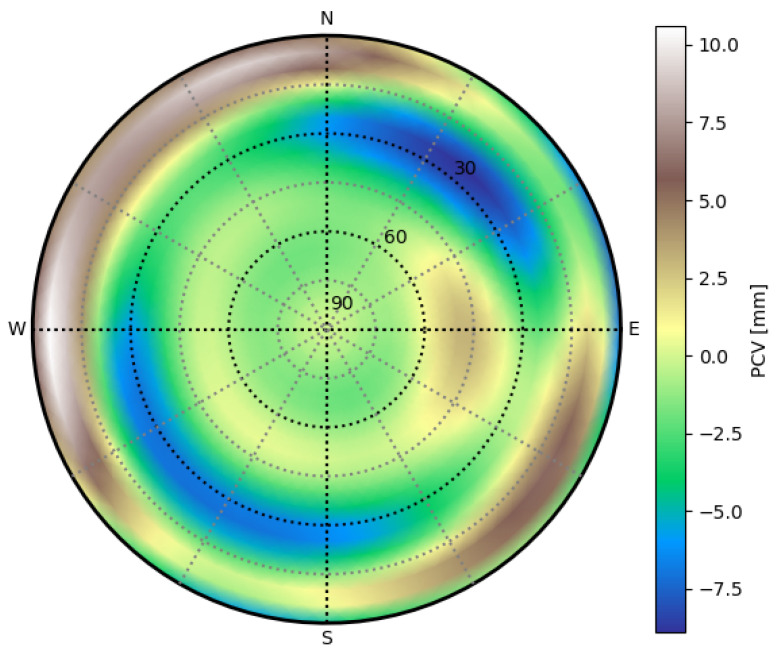
Result of the linear IF combination of both L1 and L2 GPS (Global Positioning System) PCV (Phase Center Variation) maps for Trimble TRM105000.10 antenna.

The antenna phase center variation (PCV) was simulated by adding a Trimble TRM105000.10 PCV offset to the signal. This antenna was selected because it can be mounted on R/V. The PCV effect for the Trimble antenna is found in the antenna calibration file ngs14.atx provided by the National Geodetic Survey (NGS) [61]. A cubic interpolation of the antenna calibration map has been chosen, resulting in the PCV map in Figure 1 for GPS case. Figure 1 shows the linear combination of linear IF combination PCV maps for the GPS constellation, which is suitable for modeling $PCV_{IF}$ of Equation (1).

#### 2.1.6. Multipath Effect

Our multipath effect model is inspired by the model used by King et al. [53]. In Equation (1), $MP_{IF}$ results in a linear IF combination of multipath effects $MP_{f_{i}}$ from Equation (3) computed at both GNSS frequencies $f_{i}$.

(3) $$MP_{f_{i}}=\frac{\lambda_{i}}{2\pi }\arctan\frac{a\sin\left(4\pi \frac{H_{0}}{\lambda_{i}}\sin\epsilon \right)}{g_{d}+a\cos\left(4\pi \frac{H_{0}}{\lambda_{i}}\sin\epsilon \right)}$$

where $\lambda_{i}$ denotes the wavelength of the observed signal of frequency $f_{i}$, $H_{0}$ the height of the antenna above the sea surface, ϵ the elevation of the satellite, and $a=Sg_{r}R_{a}$ the amplitude of the reflected signal on a surface of roughness S=1 such as sea water. $g_{d}=\cos z/G$ and $g_{r}=\cos\left(90/G\right)\left(1-\sin\epsilon \right)$ are the direct and reflected gain defined with the rate of change G=1.1 of the antenna gain and *z* the angle of the observed satellite to the zenith. To implement the right-hand circular polarization of the GNSS signal, the Fresnel equation implemented becomes the following:

(4) $$R_{a}=\sqrt{R_{a_{perp}}^{2}+R_{a_{para}}^{2}}$$

where

(5) $$R_{a_{para}}=\frac{-n_{2}\cos z+n_{1}\sqrt{1-{\left(\frac{n_{1}}{n_{2}}\sin z\right)}^{2}}}{n_{1}\cos z+\sqrt{n_{2}^{2}-{\left(n_{1}\sin z\right)}^{2}}}$$

denotes the parallel Fresnel coefficient, and

(6) $$R_{a_{perp}}=\frac{n_{1}\cos z-\sqrt{n_{2}^{2}-{\left(n_{1}\sin z\right)}^{2}}}{n_{1}\cos z+\sqrt{n_{2}^{2}-{\left(n_{1}\sin z\right)}^{2}}}$$

denotes the perpendicular Fresnel coefficient, where $n_{1}=1$ is the refractive index of air and $n_{2}=1.33$ is the refractive index of salt water.

**Figure 2:**
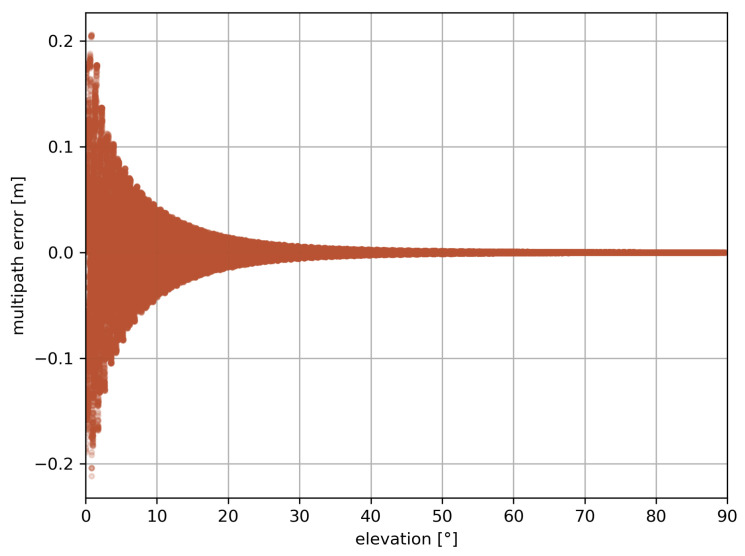
Simulated linear IF combination GNSS (Global Navigation Satellite System) multipath error for a 30-m high Trimble TRM105000.10 antenna at mid-latitudes.

Figure 2 shows the resulting linear IF combination multipath as seen from the simulated antenna over 24 h. The usefulness of an elevation cut-off angle is clearly depicted in this Figure, as the higher multipath values appear at the lowest elevations.

#### 2.1.7. Noise Measurement Simulation

The precision of the carrier phase measurement is 0.01 cycles [62]. The noise from the carrier phase measurement $\eta_{IF}$ reduced in terms of distance is then simulated as white noise modulated by a linear IF combination of wavelengths $\lambda_{1}$ and $\lambda_{2}$ of a GNSS satellite, as in Equation (7).

(7) $$\sigma_{\eta }^{2}={\left(IF1\frac{\lambda_{1}}{100}\right)}^{2}+{\left(IF2\frac{\lambda_{2}}{100}\right)}^{2}$$

where $\sigma_{\eta }^{2}$ denotes the variance applied to the white noise.

**Figure 3:**
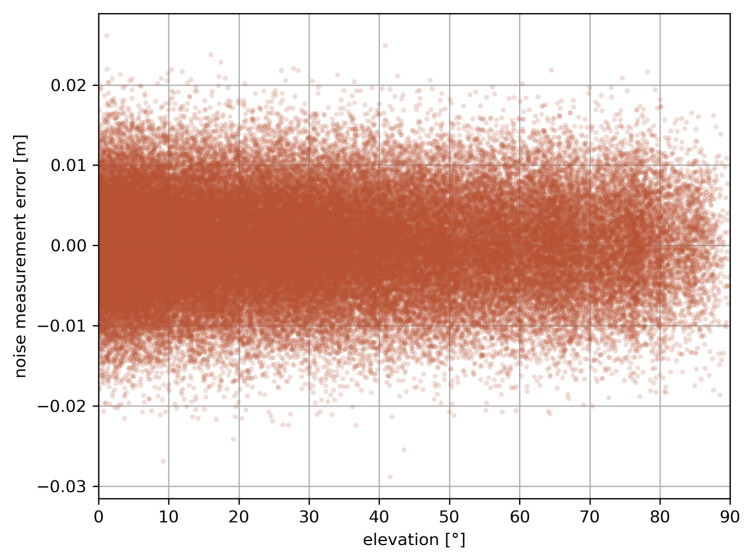
Simulated linear IF combination GNSS noise measurement error for a Trimble TRM105000.10 antenna at mid-latitudes.

Figure 3 shows the linear IF combination of the noise measurement simulated during a 24-h GNSS measurement at mid-latitudes. The magnitude of this error appears to be ten times weaker than the maximum multipath error of Figure 2, but it is evenly distributed around the skymap, contrary to the multipath that shows greater errors at low elevations than near the zenith.

### 2.2. Data Processing of Simulated GNSS Measurements

#### 2.2.1. Estimation Parameters

Different parameters such as receiver clock error, 3D positioning, ZWD, and tropospheric gradients are estimated from the processing of the carrier phase measurement simulated as in Section 2.1.1, according to Equation (8).

(8) $$\phi_{i}=\hat{C}+\hat{E}\cos\epsilon \sin\alpha +\hat{N}\cos\epsilon \cos\alpha +\hat{U}\sin\epsilon +\frac{Z\hat{W}D}{\sin\epsilon }+\frac{{\hat{G}}_{N}\cos\alpha +{\hat{G}}_{E}\sin\alpha }{\sin\epsilon \tan\epsilon }$$

where $\phi_{i}$ denotes the carrier phase observable from the i-th satellite, $\hat{C}$ is the estimated clock error, $\hat{E}$, $\hat{N}$, and $\hat{U}$ are the estimated East, North, and Up position components of the antenna, respectively, $Z\hat{W}D$ is the estimated troposphere wet delay, and ${\hat{G}}_{N}$ and ${\hat{G}}_{E}$ are the zonal and meridional estimated gradients of the troposphere. The components ϵ and α are the elevation and azimuth of the i-th satellite. This carrier phase modeling is equivalent to Equation (1), but we added the horizontal positioning of the antenna and the tropospheric gradients because they could be impacted by the simulated errors in the GNSS signal as well.

Equation (8) is linear, so the seven parameters can be estimated by processing the carrier phase measurement through a Kalman filter [63]. As the vessel carrying the GNSS antenna is moving on the sea surface, the three positioning components have low constraints over time, so we chose a high value here, set to $\sigma_{C,E,N,U}^{2}=10^{2}$ m${}^{2}$. The clock constraint has been set to the same value. The variance in ZWD is calculated from a given random walk value. The variance in tropospheric gradients is chosen a hundred times smaller than the variance of ZWD: $\sigma_{ZWD}^{2}=100\sigma_{G_{N,E}}^{2}$ [45].

The implemented Kalman filter has several features developed in the following sections that can be set up as processing options:

- the cut-off angle;
- the weighting function of the elevation of the observed satellites;
- the random walk on the ZWD.

#### 2.2.2. Cut-Off Angle

It is essential to remove signals from the satellites that are too low above the horizon of the observing antenna. It allows fewer multipath errors to affect the results.

It is also important to choose an elevation cut-off angle low enough above the horizon. A small angle elevation satellite will permit to keep more data coming from the satellites, giving more observations to the Kalman filter. Height and ZWD can also be more easily decorrelated due to the low-elevation satellites than with the satellites near the zenith of the antenna [64]. Therefore, the elevation cut-off angle is the first sensitive setting of the Kalman filter.

Here, we studied the commonly used values of 3°, 7°, and 10° of elevation cut-off angle. The cut-off angles of 3° [65] and 7° [66] have been chosen because their impact on tropospheric gradients is studied by Kačmařík et al. [49], and 10° is also being studied to process GNSS data in Meindl et al. [67]. They are also commonly used elevation cut-off angles for processing shipborne GNSS data [22,23,27,68].

#### 2.2.3. The Weighting Function

For the same reason as for the elevation cut-off angle, it is also possible to implement a weighting function applied according to the elevation of the observed satellites. This method permits taking the low elevation measurements into account but giving them less weight in the estimation process than the zenithal observations. Weighting is involved in the uncertainty of phase measurement in the Kalman filter [69,70].

The implemented processing elevation weighting functions studied here are:

- uniform, which applies a constant weighting to all satellite observations, regardless of their elevation.
- sine, which modulates the uniform weighting by dividing it by the sine function applied to the elevation of the observed satellite. In this way, the measurements coming from low-elevation satellites have greater uncertainty. They are less taken into account.
- sqrtsin, which modulates the weighting of sine by applying the square root function to the sine function of elevation.
- cos4, which modulates the uniform weighting by multiplying it by the elevation weighting proposed by Hadas et al. [46]: $\sqrt{\left(1+4\cos\epsilon^{8}\right)}$, where ϵ denotes the elevation of the observed satellite.

**Figure 4:**
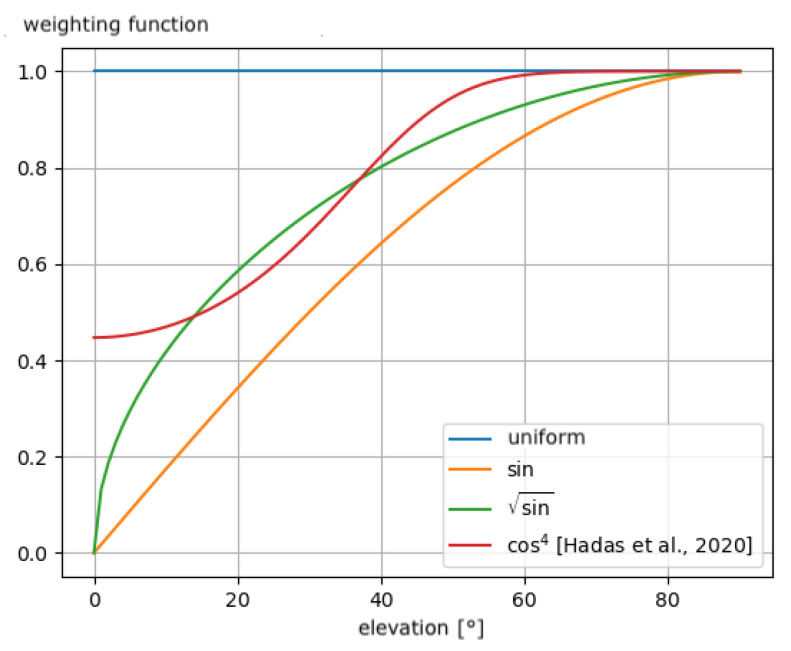
Weighting functions applied to the elevation of the satellite. They are applied to modulate the uncertainty of the carrier phase measurement. The red weighting function named $\cos^{4}$ in the legend is the weighting function $\sqrt{\left(1+4\cos\epsilon^{8}\right)}$ proposed by Hadas et al. [46].

These functions applied by dividing the measurement weighting originally uniformly set to 1 cm in the Kalman filter are shown in Figure 4.

Figure 4 shows that adding the square root to the sine function allows obtaining less measurement uncertainty than the sine function itself. The cos4 function modulates the low elevations differently than the high elevations. It gives more certainty to these low elevations than the usual weighting functions presented above. Elevations higher than 60° have uncertainty similar to the uniform weighting with this function, assuming that observations in a 30° cone around the zenith of the antenna have almost the same significance.

#### 2.2.4. Random Walk on ZWD

Variations in tropospheric components within the Kalman filtering process are constrained, so some of the carrier phase noise resulting from the height movement is not incorrectly assimilated to the ZWD component. This constraint on ZWD variations through time, called the random walk, is usually parameterizable by the user during GNSS processing, so we set it as a third processing parameter in the Kalman filter. Studies about PPP processing shipborne GNSS at sea usually use random walk process noise around 5 mm·h${}^{-1/2}$ [22,41,71], but values can be found from less than 1 mm·h${}^{-1/2}$ [68] up to 20 mm·h${}^{-1/2}$ [72].

The tested values for the random walk parameter in the following processing modeling study were 1, 3, 5, 7, 8, 10, 12, 15, and 20 mm·h${}^{-1/2}$.

### 2.3. Features of the Reference Study

A first study has been carried out with some characteristics explained in Section 2.3.1, Section 2.3.2, Section 2.3.3 and Section 2.3.4 of which the main are listed in Table 2. This study is called "reference study" in the following.

#### 2.3.1. GNSS Constellations Used for the Reference Study

The satellite constellations observed for the reference study were GPS, GLONASS, and Galileo. We selected a multi-GNSS study because it usually yields better results than using only the GPS constellation [73]. Indeed, using more constellations enables more satellites to be observed at each time and better geometry, so variables should be estimated more precisely.

#### 2.3.2. Data Sampling for the Reference Study

The time resolution chosen for the processing of the reference study was set to 30 s, as it is the resolution used in previous work on real GNSS data processing parameters [50], and commonly used when processing a shipborne GNSS antenna [22,41,74]. It also allows limiting the processing duration compared to 1 s time resolution.

#### 2.3.3. Shipborne Antenna Location and Movements for the Reference Study

To avoid simulating the displacement of the vessel, we assumed that the vessel stayed at the same longitude and mean altitude. We set them up, respectively, to 0° and MSL during the whole 24 h acquisition simulation. We fixed the latitude to 45° N for the reference study. It corresponds to mid-latitudes, like those of France, where data from the study by Panetier et al. [50] were collected to retrieve ZWD from a real GNSS antenna.

The antenna is supposed ship-mounted 30 m above the sea surface. The tide effect applied to the MSL is modeled with a 12 m peak-to-peak amplitude sine function with a 12 h period. The heave is modeled by a 0.4 m amplitude sine function with a period of 16 s which could correspond to a calm sea state with light wind in the summer. No changes are modeled due to the loading of the vessel (fuel, passengers, etc.), but this could affect the height of the antenna. However, it is a slow motion and we can consider that slow vertical motions are already studied within the tide modeling.

#### 2.3.4. Simulated Tropospheres for the Reference Study

The simulation provides 200 simulated tropospheres. This number is optimal, so the statistics on the estimated variables show little bias compared to a smaller number of simulations, but we noticed no significant changes when increasing the number of tropospheres. The 200 reference study ZWD are simulated with a 5 mm·h${}^{-1/2}$ random walk variation, which is a common tropospheric value at mid-latitude [75].

#### 2.3.5. Antenna PCV for the Reference Study

The PCV of the antenna that affects the measurement of the GNSS signal is supposed to be perfectly corrected for the reference study. To simplify the simulation we did not add any PCV effect to the simulated carrier phase, which means that $PCV_{IF}\left(\alpha ,\epsilon \right)=0$ mm. Consequently, during the estimation, no correction is applied either.

### 2.4. Further Investigation

From the reference study, we conducted a complementary survey on the acquisition setting. The aim is to study the impact of the choice of the constellations used, the time resolution, and the location of the carrying R/V on the data processing results. Each of these three aspects constituted a new study with all other acquisition settings than the one studied similar to the reference study of Section 2.3. We also studied the PCV effect of the antenna and its correction. Table 3 describes the acquisition parameters of the reference study and the other parameters under study during these investigations. These characteristics are explained in Section 2.4.1, Section 2.4.2, Section 2.4.3 and Section 2.4.4.

#### 2.4.1. GNSS Constellations

To evaluate the contribution of multi-constellation use compared to only one constellation, we conducted another study of the processing parameters on GPS signals only, from the reference simulation.

#### 2.4.2. Data Sampling

We studied the impact of time resolution considering only one datum out of ten within the reference study during another processing. This results in a new estimation with a 300 s time resolution instead of 30 s. We will then be able to discuss the importance of the time resolution choice.

#### 2.4.3. Latitude

The impact of the location of the R/V carrying the GNSS antenna on the processing results is studied by changing latitude. Two new simulations have been carried out with a +10° N and +80° N latitude, respectively.

The latitude of the antenna has an impact on the geometry distribution of the satellites above the antenna and it also affects the water vapor content variations in the troposphere [75].

For this reason and to adapt the simulation to this situation, we implemented simulations of 200 new tropospheres with a random walk of 2 mm·h${}^{-1/2}$ at a polar location of 80° of latitude, and 10 mm·h${}^{-1/2}$ at an equatorial location of 10° of latitude.

#### 2.4.4. PCV Correction

Different antenna orientation effects have been implemented for the PCV simulation and are studied here:

- an antenna that is always well oriented to the North, such as a well-positioned ground-based antenna or a vessel going constantly northward;
- an antenna that has a constant bias of orientation from the North, such as a constant heading vessel;
- an antenna that is moving through time, such as the boat turning around, performing a 360° over 24 h.

The estimator can correct the antenna PCV effect by three different means listed below, by adding the antenna correction to the carrier phase measurement before the Kalman filtering:

- no PCV correction is applied at all;
- the PCV correction is applied considering that the antenna is well oriented toward the North;
- the azimuthal mean of the PCV map in Figure 1 has been computed, resulting in an elevation-dependent correction of the PCV only.

The PCV values implemented for simulation and correction purposes are from the Trimble antenna presented in Section 2.1.5.

This additional study proposes a correction of the antenna PCV effect that would be suitable for a shipborne antenna whose orientation is unknown because of the displacement of the antenna. Only the antenna yaw is studied here, but implementing the other angular movements would have been interesting to study their effects.

## 3. Results

The results presented here will focus on the height and ZWD estimation match with the simulated quantities, as well as the correlation between both estimated variables.

### 3.1. Analysis of the Solution from the Reference Simulation

#### 3.1.1. Sensitivity of Height and ZWD Mean Correlation Coefficient to Processing Modeling

**Figure 5:**
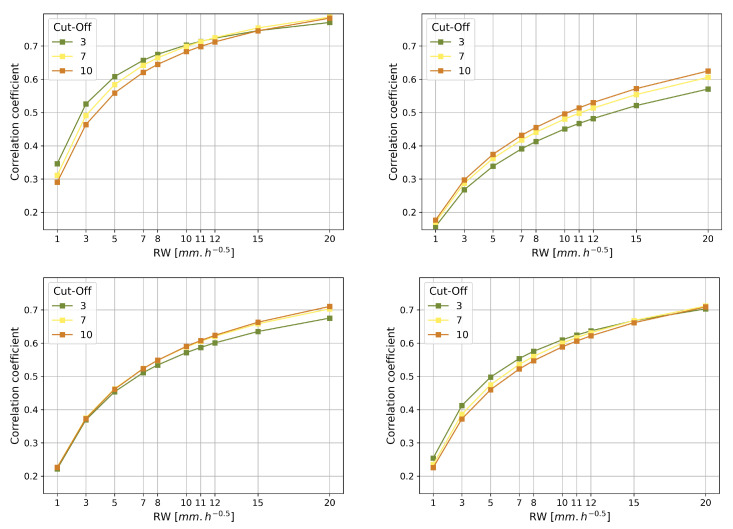
Mean correlation coefficient between height and ZWD (Zenith Wet Delay) as a function of random walk on ZWD for each cut-off angle for the elevation weighting functions uniform (**top left**), sine (**top right**), sqrtsin (**bottom left**), and cos4 (**bottom right**).

The correlation coefficient between height and ZWD has been calculated for each of the GNSS signal processing models in the 200 different tropospheres. The mean height and the ZWD correlation coefficient were then calculated from the 200 resulting correlation coefficients for each processing model. Therefore, there remains a mean correlation coefficient between height and ZWD and its standard deviation for each of the processing models. The standard deviations of the correlation coefficients are all lower than 0.1. In the following, we will focus on the mean correlation coefficient between ZWD and height presented in Figure 5.

As expected, Figure 5 shows that the mean correlation coefficient increases with the cut-off angle when using elevation weighting function modelings sine and sqrtsin, which corroborates the fact that height and ZWD are better decorrelated when embedding the signals coming from the low elevation satellites above the horizon.

However, we observe the opposite behavior on correlation and height estimation with uniform and cos4 elevation weighting functions. As shown in Figure 4, both uniform and cos4 give more weight to measurements of very low elevation angles than sine and sqrtsin. cos4 weighting reaches sqrtsin around 7° of elevation angle, and the weight of cos4 is twice as high as sqrtsin at 3° of elevation angle.

Figure 5 also shows that the mean correlation coefficient between height and ZWD is lower for low random walks. The lowest mean correlation coefficient is 0.16 obtained for 3° of cut-off angle, sine weighting function, and 1 mm·h${}^{-1/2}$ of random walk. The highest mean correlation coefficient is 0.78 for 10° of cut-off angle, uniform weighting function, and 20 mm·h${}^{-1/2}$. The function sine is the best weighting for decorrelating height and ZWD, while uniform appears to be the worst. sqrtsin appears to decorrelate height and ZWD slightly better than cos4 at 7° and especially at 3° of cut-off angle, and both at 10° of cut-off angle give the same correlation coefficient. We can notice that the change in random walk value impacts the correlation coefficient more than the cut-off angle. This might be because a tight constraint on ZWD will force this variable to absorb a limited amount of errors coming from the antenna movement, particularly the change height that could be wrongly assimilated to ZWD in the case of loose random walk value. The height part thus wrongly assimilated to the ZWD could be responsible for its correlation being increased with the height estimate.

#### 3.1.2. Sensitivity of Height and ZWD Biases to Processing Modeling

**Figure 6:**
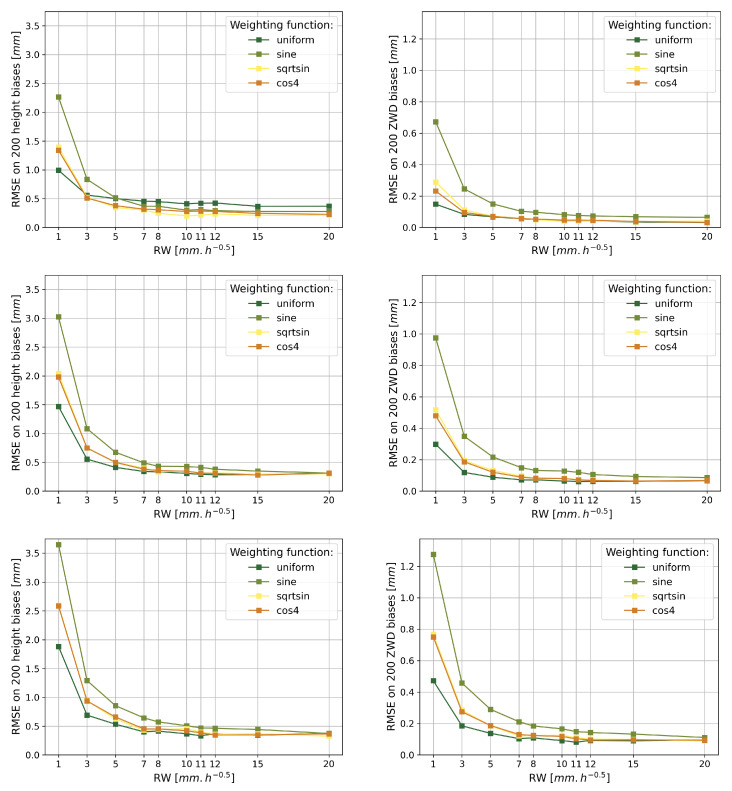
Height (**left column**) and ZWD (**right column**) root mean squared errors (RMSE) on the average of 200 different tropospheres biases between simulation and estimation, as a function of random walk on ZWD for each weighting function at a cut-off angle of 3° (**top**), 7° (**center**) and 10° (**bottom**).

The biases between the estimation and the simulation on height and ZWD have been calculated for each of the GNSS signal processing models over the 200 different tropospheres. The average height and ZWD biases were then calculated from the 200 resulting biases for each processing model, as well as the associated dispersion. From this, the root mean square error (RMSE) of the height bias and ZWD have been calculated for each of the processing models and are presented in Figure 6.

Figure 6 shows that, despite the large amplitude of the simulated vertical movements, the height and ZWD estimates are still of good quality. Indeed, the height bias RMSE is 3.7 mm at most, with (−0.3±3.7) mm of height bias obtained for 10° of cut-off angle, 1 mm·h${}^{-1/2}$ of processing random walk and sine weighting function. This processing modeling also gives the highest ZWD bias RMSE of 1.3 mm, with (−0.1±1.3) mm of ZWD bias. The major part of the highest RMSE values on biases is due to the dispersion of the biases being greater than the average. Indeed, the bias averages in the ZWD estimation are less than 0.1 mm, except when using a cut-off angle of 10°, which occurs with an average ZWD bias of at most 0.2 mm for all weighting functions but sine. Furthermore, average height biases range from 0.0 mm to 0.2 mm when using a cut-off angle of 3° or 7°, except when using uniform weighting function occurring at a maximum height bias average of 0.4 mm with 3° of cut-off angle. For 10° of cut-off angle, the average height biases range from 0.3 mm to 0.6 mm, depending on the weighting function and random walk modeling.

Figure 6 also shows that increasing the random walk value in the ZWD will decrease the RMSE in the biases for height and ZWD for any given cut-off angle and weighting function modeling. In light of the elements presented in the previous paragraph, this shows that releasing the constraint on the ZWD will allow us to obtain more precision on the biases between simulation and estimation of height and ZWD. This result corroborates the observations made on ground antennas by Young et al. [76], recommending the use of at least 6 mm·h${}^{-1/2}$ random walk on ZWD to process the GNSS data.

Decreasing the cut-off angle allows us to decrease the RMSE according to the three graphs for both variables in Figure 6, which is logical considering the fact that a low cut-off angle allows decorrelating height and ZWD. However, this result is due to the fact that the multipath effect is quite low in this shipborne antenna simulation, requiring a well-placed antenna, for example, at the very top of the vessel. Bosser et al. [27] showed that some antenna locations are not suitable for retrieving ZWD from shipborne antennas, as the quality of ZWD can be degraded even when using a cut-off angle greater than 3°. Thus, it will be preferred to find the most suitable location as possible for the GNSS antenna onboard the vessel to prevent the multipath effect, and then process the data with a low cut-off angle to well decorrelate height and ZWD.

The weighting function sine appears to give the worst RMSE, while the weighting functions cos4 and sqrtsin have a similar behavior both in the resulting height and ZWD estimates. Indeed, if sine systematically gives the best average bias on both variables, it appears that the dispersion of the ZWD bias is up to five times worse than when using the other weighting functions, while it is up to three times worse in the height bias dispersion. uniform seems to be the best estimation weighting function model, except to estimate the height at 3° of cut-off angle. However, it is worth mentioning that the uniform function provides less accuracy than the other weighting models since it gives a systematically worst average of the bias on both height and ZWD, which can be up to three times higher on ZWD and four times higher on height than with any other weighting function.

#### 3.1.3. Sensitivity of Height and ZWD Standard Deviations to Processing Modeling

The standard deviation of the difference between the estimates and the initial simulation for ZWD and height has been calculated for each of the 200 different tropospheres simulated with each processing modeling. The average of the 200 standard deviations of the difference between the estimation and the simulation of height and ZWD has then been computed for each processing modeling, as well as the associated dispersion.

First, Table 4 presents some of the dispersion figures of the standard deviations of the differences between the estimation and the simulation of height and ZWD.

The dispersion figures in Table 4 are very low, most of the time lower than 1 mm, and even lower than 0.5 mm on ZWD. The highest dispersion is always displayed for the weighting function sine, implying more variability in the accuracy of the height and ZWD estimation when using this elevation weighting modeling, compared to the other weighting functions tested. It appears that using low random walk values or increasing the processing cut-off angle increases the variability of the standard deviations on height and ZWD differences, for any elevation weighting function. In light of this very small dispersion of the standard deviation of the differences, the following will focus on the average of the standard deviation of the differences between the estimation and the simulation of height and ZWD.

**Figure 7:**
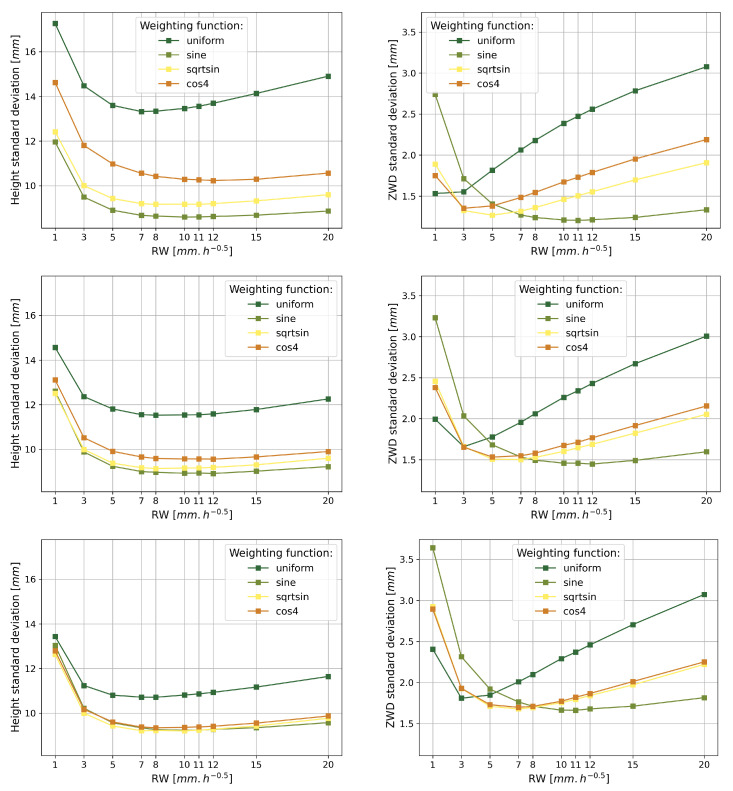
Average of the standard deviations of the difference between estimation and simulation of height (**left column**) and ZWD (**right column**) over the 200 different simulated tropospheres as a function of random walk on ZWD for each weighting function at a cut-off angle of 3° (**top**), 7° (**center**), and 10° (**bottom**).

The averages of the standard deviations for height and ZWD are presented in Figure 7.

Figure 7 shows the averages of the standard deviation of the differences in ZWD and height as a function of the processing random walk, for each weighting function (different colors) and cut-off angle (different graphs) tested. In each plot, the use of a uniform weighting function worsens the average standard deviation compared to the other weighting functions. The only exception is when processing with very low processing random walks, where uniforms performs better at estimating ZWD, but still provides the worst height estimate of all weighting functions. Then, it appears that setting up a weighting function on the elevation of the observed satellites helps to improve the estimation of height and ZWD during the GNSS PPP signal processing instead of giving the same weighting to all observations.

The weighting function sine systematically provides us with the lowest ZWD average standard deviation for any elevation cut-off angle modeling. The minimum value of the ZWD average standard deviation is obtained for random walks in the range of 10 to 12 mm·h${}^{-1/2}$, depending on the cut-off angle. Then it appears that the ZWD average standard deviation is better when using higher random walk values than in the simulation. As an approximation, we can state that the sine function needs to loosen the random walk twice to minimize the standard deviations of the differences.

The weighting functions cos4 and sqrtsin exhibit behavior similar to each other, especially as the cut-off angle increases. However, the use of the weighting function cos4 always induces a higher standard deviation than sqrtsin for height estimates. Furthermore, only in the case of a random walk lower than 3 mm·h${}^{-1/2}$ the weighting function cos4 achieved a better estimation of ZWD than sqrtsin. Finally, the graphs of the average standard deviation on the height difference processed with the weighting functions uniform and cos4 in Figure 4 show that increasing the cut-off angle improves the height estimation with both weighting models. This might be explained by the multipath effect, as discussed in Section 3.1.4.2. Therefore, we chose to focus on the impact of sqrtsin and sine.

Table 5 shows the minimum values of the average standard deviation for ZWD and height differences for the three tested cut-off angles and the sqrtsin and sine weighting functions.

It is noticeable that all the standard deviation minima presented in Table 5 are not obtained with the same random walk value on the ZWD. Therefore, the last column of Table 5 presents the random walk values that provide the minimum standard deviation on ZWD and height for each pair of modeling on the cut-off angle and weighting function.

The average standard deviation shown in the fourth column of Table 5 shows that the configuration that gives the closest ZWD and height estimates to the reference is 3° of cut-off angle and weighting function of elevation sine with a random walk of 11 mm·h${}^{-1/2}$. The rate of change shows that the increase in the cut-off angle induces an increase in the standard deviation of the ZWD differences up to +20.3% for 7° and +38.2% for 10°. The height results also show that increasing the cut-off angle decreases the resulting minimum in the average standard deviation of the height difference, with a change rate of +3.7% for 7° and +7.4% for 10°.

At a given cut-off angle, the minimum average standard deviation in ZWD is obtained using the weighting function sine instead of sqrtsin. However, the differences remain very low, in the 0.1 mm range, and tend to decrease when the cut-off angle increases. For a cut-off angle of 3°, the minimum standard deviation obtained by using the sqrtsin weighting function is +5.3% higher than when using the function sine. At 7°, the rate of change of sqrtsin is +3.8% higher than sine and decreases to +0.9% at a cut-off angle of 10°. The same conclusion is reached when considering the height estimates. A +6.6% increase in the average standard deviation of the difference is obtained when using sqrtsin compared to sine for a cut-off angle of 3° and +2.5% at 7°. The function sqrtsin even provides a +0.3% better average standard deviation minima than sine for a cut-off angle of 10°. It appears that the choice of weighting functions between sine and sqrtsin mainly affects the low-elevation satellite signals; then it decreases with increasing cut-off angle [46].

Table 6 further details the ZWD estimate for each of these random walks. The three last columns of Table 6 show that the sqrtsin function is 9.8% better at estimating ZWD than the function sine at a cut-off angle of 3°, with the 5 mm·h${}^{-1/2}$ random walk corresponding to the simulated ZWD. At a minimum, obtained with a cut-off angle of 3°, sine weighting function, and 11 mm·h${}^{-1/2}$ of random walk, sqrtsin degrades the estimate of ZWD by +28.2%. In general, the rate of change between sqrtsin and sine decreases when the cut-off angle increases, but the ZWD standard deviations also increase compared to a cut-off angle of 3°.

The use of the weighting function sine implies the need to double the random walk value for a better estimation compared to the simulation. The weighting function sqrtsin appears to work well with lower random walks, such as 5 mm·h${}^{-1/2}$ for 3° of cut-off angle and 7 mm·h${}^{-1/2}$ for higher cut-off angles, instead of 11 to 12 mm·h${}^{-1/2}$ for obtaining the minimum standard deviation with the weighting function sine. As ZWD was simulated with a 5 mm·h${}^{-1/2}$ random walk, sqrtsin seems to remain consistent with the physics of atmospheric water vapor compared to the function sine. Indeed, Table 7 shows that for the three cut-off angle values tested, the sqrtsin function performs around +10% better in estimating the ZWD than sine weighting function when using 5 mm·h${}^{-1/2}$ of random walk modeling. The height estimation with sqrtsin at 5 mm·h${}^{-1/2}$ does not change with the elevation cut-off angle. However, the function sine performs better at a very low elevation cut-off angle, showing an improvement of +5.9% at most for 3°. Both weighting functions give similar results to the rate ±1.5% for the other two cut-off angles.

#### 3.1.4. Highlight of a Suitable Processing Modeling

##### 3.1.4.1. Cut-Off Angle

Regarding the cut-off angle, Figure 5 shows that as expected (Section 2.2.2), in the case of a sine or a sqrtsin weighting function modeling, decreasing the cut-off angle decreases the correlation between height and ZWD. This is consistent with the real data study [50] giving the best results when using low cut-off angles.

On the other hand, Figure 6 and Figure 7 show that the increase in cut-off angle significantly degrades the accuracy of the height and ZWD estimations and the precision of the ZWD estimation, for any weighting function. Figure 7 shows in turn that interestingly the height precision is improved for high cut-off angles with uniform and cos4 weighting functions, whereas the height precision is very slightly improved for low cut-off angles with the weighting function sine and is not affected by the cut-off angle choice with sqrtsin weighting function.

##### 3.1.4.2. Elevation Weighting Function

As depicted in Section 3.1.4.1, the effect of the elevation weighting function is closely related to the applied cut-off angle. Taking into account the height graphs in Figure 7, the weighting function cos4 shows significant deterioration of the height at 3° of cut-off angle compared to 7° and 10° of cut-off angles; the fact that there is almost no change in height precision when changing the cut-off angle, 3° of cut-off angle even providing the best estimation precision in both cases; and the fact that in Figure 7, cos4 appears to systematically deteriorate height estimation compared to sine and sqrtsin and that uniform is even worse in estimating height and ZWD; we can then conclude that if the low elevation data are useful for precisely processing the GNSS measurements, weighting them with an appropriate elevation function is a key processing modeling to obtain better height and ZWD estimations.

Figure 5 shows that the elevation weighting functions uniform and cos4 both have the expected increase in the correlation coefficient with the cut-off angle only for 20 mm·h${}^{-1/2}$ of random walk, but curiously show the contrary for the lower random walks. This could be because the multipath affects mainly low elevation signals, which are given more weight in the case of the elevation weighting functions uniform or cos4 (Figure 4). Therefore, and especially at a low cut-off angle, the elevation weighting functions sine or sqrtsin will be preferred to correctly process the data despite the effect of the multipath during the estimation process. A study on real data [50] showed the best results when using sqrtsin as well, but the weighting function uniform was also satisfying contrary to the simulation results.

##### 3.1.4.3. Random Walk on ZWD

Section 3.1.3 shows that the standard deviation of the 200 differences between estimation and simulation for each processing model is low on average, but its variation is significant and shows a convex shape in Figure 7 for the height and ZWD estimates as a function of random walk. This means that there is a nominal random walk value to use to process the GNSS data passing through a 5 mm·h${}^{-1/2}$ ZWD varying simulated troposphere.

Section 3.1.2 shows that the elevation weighting function sine degrades the accuracy of height and ZWD estimation compared to sqrtsin and cos4. uniform appears to be the best performing weighting function, except with a low cut-off angle of 3° where it is resulting in the worst height estimation for random walks higher than 5 mm·h${}^{-1/2}$. On the face of it, to accurately estimate height and ZWD we recommend favoring weighting functions cos4 or sqrtsin when using GNSS measurements at low elevation angles with a random walk value around 5 mm·h${}^{-1/2}$ or above. This is consistent with the best resulting processing of real data from Panetier et al. [50], obtained for a random walk model of 3 to 5 mm·h${}^{-1/2}$.

As seen in Section 3.1.1, the correlation coefficient for 3° of cut-off angle and 5 mm·h${}^{-1/2}$ of ZWD random walk is 0.45 with sqrtsin as a weighting function in elevation, while it is as low as 0.33 when using the weighting function sine. However, the function sine also shows a correlation coefficient of 0.45 as the previous sqrtsin with its nominal random walk value of 10 mm·h${}^{-1/2}$ at 3° of cut-off angle.

Young et al. [76] have observed that processing GPS PPP data with GipsyX software (JPL, NASA [77]) requires increasing the random walk to at least 6 mm·h${}^{-1/2}$ from the default 3 mm·h${}^{-1/2}$ random walk on ZWD. This could be explained by the fact that the data were processed using the weighting function sine, which requires a higher random walk to detect the variability of ZWD, according to the above results. The same study could then be conducted using the sqrtsin weighting function to validate the simulation results and to try to acquire a suitable GPS positioning through a more accurate physical model.

Selle et al. [48] have presented a similar observation. They recommend an 8.4 mm·h${}^{-1/2}$ of random walk when using a sine weighting function, whereas a 5.4 mm·h${}^{-1/2}$ is sufficient when processing with sqrtsin weighting function for the same ground GNSS antenna data set. Then it seems that the shipborne simulation of the GNSS antenna corroborates these ground antenna observations with a rise in processing the random walk needed when using the sine weighting function.

sqrtsin weighting function then seems to be a decent compromise to achieve the best ZWD possible for meteorology purposes while staying coherent with the physical process. It is important to keep in mind that at low elevation cut-off angles, this weighting function does not provide the optimal height as a sine function will likely provide a slightly better height estimate.

##### 3.1.4.4. Recommended Modeling

For the rest of the study, we then selected a configuration to process GNSS data for meteorology purposes, of a shipborne antenna placed at the highest point of the vessel: 3° of cut-off angle, sqrtsin as a weighting function on the elevation, and 5 mm·h${}^{-1/2}$ of ZWD random walk. This processing provides an RMSE of the difference between the estimates and the simulation of 9.4 mm on the height and 1.3 mm on the ZWD for this reference study.

It is important to notice that such as explained in Section 2.1.4, the mapping function used to map the ZWD to the simulated signal path is a simple sine model, and the same model is used to compute the ZWD from the signal path through the troposphere. However, a tropospheric mismodeling has been shown to impact the accuracy of the troposphere estimation up to 1 mm [78]. Modeling this error as well as random walk, cut-off, and measurement weighting could have been interesting in our simulations, and further work should be carried out in order to evaluate its impact on the processing model. In the following, the tropospheric mapping model is considered correct.

### 3.2. Investigation of Other Analysis Parameters

#### 3.2.1. Data Sampling

We investigate the impact of data sampling on the estimation of ZWD and height. Thus, we consider downsampling GNSS raw data to 300 s instead of 30 s as in the reference simulation. This lower-resolution PPP processing leads to a degradation of the estimates. In fact, with a modeling of 3° of cut-off angle, sqrtsin weighting function and 5 mm·h${}^{-1/2}$, the average standard deviation of the difference between estimates and simulation increased by +11.9% in height and +43.9% in ZWD as shown in Table 8.

The decrease in resolution creates an increase of +37% in the correlation coefficient for the selected processing configuration.

We can then conclude that using high-resolution data sampling is important for obtaining better results on a PPP processing of shipborne antennas.

#### 3.2.2. GNSS Constellations

Unlike the reference simulation performed with three constellations (GPS, GLONASS, and Galileo), we modified the reference simulation by considering only GPS observations. This change impacts the number of data available at each processing time and the geometry of the observed satellites. Using only GPS satellites induces a degradation of the estimation. Indeed, the average standard deviation increases by +59.8% for the height estimate and +51.1% for the ZWD for a cut-off angle of 3°, weighting function sqrtsin, and 5 mm·h${}^{-1/2}$ of random walk, as shown in Table 9.

GPS-only height bias is almost twice better than multi-constellation with uniform weighting function and 5 mm·h${}^{-1/2}$ but still is not better than the selected configuration for the reference simulation. The multi-constellation processing is better at estimating the ZWD than the GPS-only processing in all the configurations tested.

Then, if several constellations are available, it is very important to use all of them to reduce the estimation error, with an improvement that can reach a factor of 2. On the other hand, the mean correlation coefficient between ZWD and height is slightly better when using only GPS, with an improvement of +12.8% compared to multi-GNSS processing. This effect seems to be contrary to common sense, so it might be the first evidence of some weaknesses of our simulation.

#### 3.2.3. Antenna Phase Center Variation

The impact of the PCV of the receiver antenna has been added to the reference simulation of GNSS observation. Three different movements of the boat have been simulated:

- To North: the vessel is heading North all the time during the 24 h of the simulation;
- 45° North: the vessel is heading 45° towards the West during the whole time;
- 24-h turn: the vessel is turning at constant speed, completing a 360° turn in 24 h.

These three simulated observation sets have been processed using three different ways of correcting the antenna effect before the estimation of the variables:

- No correction: the antenna effect is not corrected, so it still affects the signal during estimation;
- North correction: the PCV correction is applied to the signal assuming that the antenna was well oriented to the North, as would have been the case with a fixed ground GNSS antenna;
- Azimuthal mean: the PCV correction applied is an azimuthal mean of the antenna PCV map so that the correction depends only on the elevation of the observed satellite.

Table 10 summarizes the average ZWD and height biases obtained with each of the PCV antenna configurations, for the selected processing parameters of 3° of cut-off angle, sqrtsin weighting function and 5 mm·h${}^{-1/2}$ of random walk. The North correction of the antenna PCV when heading North does not apply as it corresponds to the reference simulation. The absence of antenna correction induces a height bias of almost 1 cm depending on the displacement of the vessel. The bias on ZWD is lower, around 1 mm. The application of the North correction reduces these biases, with a decrease in the bias to around 1 mm on height and 0.2–0.3 mm on ZWD. The North correction is slightly better than the azimuthal mean correction when the heading is constant. This means that the heading of the vessel affects the estimation in the case of a linear path with an azimuthal correction. However, the azimuthal mean correction performs well in correcting the bias of the PCV antenna for more complicated vessel displacements, such as a turn over 24 h. The biases are then decreased to 0.1 mm on both height and ZWD.

Table 11 shows the change in the average standard deviations of height and ZWD for each antenna configuration and correction, compared to the reference simulation, using the reference modeling configuration. Without correction of the antenna effect, the average standard deviation increases by less than +10% on height up to +22% on ZWD for the turning antenna. Applying a correction using a North-oriented PCV map lowers the average standard deviation in height and ZWD. Contrarily to the bias, in all the cases both average standard deviations improve when applying an azimuthal mean correction. The azimuthal mean correction then appears to be a good alternative to correct the antenna PCV to balance the unknown changing horizontal orientation of the antenna through time.

#### 3.2.4. Zonal Displacement of the Antenna

Here, we study the impact of the change in the latitude of the R/V.

Now we consider the simulation of 200 tropospheres using different locations than the mid-latitude of the reference processing.

The multipath has been computed for these latitudes, as the geometry of the satellites above the antenna changes depending on the latitude of the location. At high latitudes, the satellites will stay at low elevations, whereas at low latitudes, the satellites will cover the entire sky above the antenna.

Estimating the ZWD and height at these latitudes with 3° of cut-off angle, sqrtsin weighting function, and 5 mm·h${}^{-1/2}$ of random walk resulted in an increase in the average standard deviation of height of +29.2% and +9.7% for latitudes of 10° and 80°, respectively, as shown in Table 12 and Table 13. The ZWD average standard deviation increases by +48% at 10° of latitude and decreases by −10.9% at 80° of latitude. It is worth noting that the estimation with 10 mm·h${}^{-1/2}$ of the 10° of latitude signal gives an increase in the ZWD average standard deviation of only +23%, which is half the increase with processing at 5 mm·h${}^{-1/2}$. The decrease at latitude 80° hits −31% with processing at 3 mm·h${}^{-1/2}$ of random walk, corresponding better to the simulated signal. Then it appears that using a suitable random walk for the region of study allows for twice as good results as keeping a medium value by default. However, it may be difficult to adapt the random walk processing to the region where the vessel operates, as it is moving all the time, and the mean random walk depends on seasons and years and is not uniform at a given latitude [75].

Changing the latitudes does not appear to have a significant effect on the mean correlation coefficient.

A more chaotic ZWD is more difficult to predict. This may explain why the results are better for low random walk simulated GNSS signals. It is relevant to use a processing random walk as close as possible to the ZWD variations at the location of the study. If possible, models or reanalysis could be used to determine an approximate value of the random walk ZWD at the antenna location. However, 5 mm·h${}^{-1/2}$ gives suitable results for all latitudes, so this mean value can be an adequate compromise for processing at all latitudes.

## 4. Conclusions and Perspectives

An accurate estimation of tropospheric delay is essential for the analysis of kinematic PPP GNSS data such as those acquired from shipborne antennas. This is especially critical as the troposphere delay is highly correlated with the height during the estimation process. In this study, we simulated shipborne GNSS measurements to assess the impact of the modeling strategy on the estimation. We applied a Kalman filtering of the simulated GNSS measurements to highlight a standing-out processing configuration for PPP shipborne GNSS.

The results corroborate previous observations made on a real dataset under simple acquisition conditions [50]. Indeed, in this earlier study of processing parameters, the vessel was navigating into a harbor and was mainly staying docked. The present study on simulations allows us to reveal the same effect of the processing parameterization for larger vessels in the middle of the sea. The recommended shipborne GNSS PPP processing modeling from these simulations requires 3° of cut-off elevation angle, weighting function as sqrtsine of the elevation, and 5 mm·h${}^{-1/2}$ of random walk on ZWD estimation.

The use of a low cut-off angle such as 3° is shown to provide rather conclusive results, since the use of low elevation measurements permits decorrelating ZWD and height estimates, then improving the whole estimation process.

Although the weighting function sine gave, slightly, the best estimates of ZWD and height, it appears that sqrtsin better describes the physical behavior of the ZWD. Indeed, the processing random walk needs to be raised compared to the simulation, to obtain the best ZWD estimate with the sine function. The sqrtsin function then appears to be the best compromise between representing physical processes and obtaining a suitable estimation in PPP processing.

The use of a sqrtsin weighting function will require the use of a random walk of 5 mm·h${}^{-1/2}$ for a far-moving vessel. Using a random walk corresponding to the ZWD variations in the study zone will provide a better estimation. However, the use of a sine function will require doubling the random walk to obtain a better estimation, according to the simulation results. We will then require to use the sqrtsin function.

The processing of the simulated shipborne GNSS gives a +22% better estimation of the ZWD when using GPS, GLONASS, and Galileo, instead of GPS only. It is worth processing all the available constellations to obtain a broader dataset and a wider satellite geometry.

If the time-dependent orientation of the antenna is not known, we also recommend applying an azimuthal mean antenna PCV correction, instead of the usually applied PCV map. As the vessel does not face North, such as a ground GNSS antenna, this method allows adaptation of the antenna effect correction to any heading of the boat, improving the ZWD estimate of +7% compared to the usual PCV correction.

Finally, reducing the temporal resolution gives better results in the estimation. We would then recommend using at most 30 s of resolution for processing the shipborne GNSS data.

The whole simulation relies on a simplified model of the shipborne GNSS carrier phase measurement. For example, no horizontal movement of the boat was simulated. This could be implemented for further study.

In future work, the recommended processing modeling will be applied for the routine analysis of the GNSS raw data acquired by R/V from the French Oceanographic Fleet. This analysis will likely result in an estimate of the ZWD over the course of the boat, suitable for studying the space and time distribution of atmospheric water vapor over open seas.

## Figures and Tables

**Table 1:** Available frequencies used in the linear ionosphere-free (IF) combination according to the constellation of the observed satellite.

Constellation	$f_{1}$	$f_{2}$
	[MHz]	[MHz]
GPS	1575.42	1227.60
GLONASS	1602.00	1246.00
Galileo	1575.42	1207.14

**Table 2:** Setup for the reference study.

Parameter	Value	Section
Constellations	GPS, GLONASS, Galileo	Section 2.3.1
Time resolution	30 s	Section 2.3.2
Location	Mid-latitudes	Section 2.3.3
Antenna height	30 m above MSL + tide + heave	Section 2.3.3
ZWD random walk	5 mm·h${}^{-1/2}$	Section 2.3.4
Antenna PCV	Perfectly corrected	Section 2.3.5

**Table 3:** Other acquisition settings under study.

Parameter Investigated	Reference Study	Other Tested Setting	Section
Constellations	GPS, GLONASS, Galileo	GPS only	Section 2.4.1
Time resolution	30 s	300 s	Section 2.4.2
Location (ZWD random walk)	Mid-latitudes (5 mm·h${}^{-1/2}$)	Pole (2 mm·h${}^{-1/2}$) Equator (10 mm·h${}^{-1/2}$)	Section 2.4.3
Antenna orientation	None	Oriented toward North Oriented toward 45° Runs in circles in 24 h	Section 2.4.4
PCV correction	Perfectly corrected	None North-oriented Azimuthal mean	Section 2.4.4

**Table 4:** Dispersion of height and ZWD standard deviation over 200 tropospheres for different processing modelings.

Processing Modeling		Deviation of Standard Deviation of Differences [mm]							
Cut-Off	Random Walk	Height				ZWD			
[°]	[mm·h${}^{-1/2}$]	unif.	sine	sqrtsin	cos4	unif.	sine	sqrtsin	cos4
3	1	0.4	0.8	0.5	0.4	0.1	0.3	0.2	0.1
	3	0.2	0.3	0.2	0.2	0.0	0.1	0.1	0.1
	5	0.1	0.2	0.2	0.2	0.0	0.1	0.1	0.0
	10	0.1	0.1	0.1	0.1	0.0	0.1	0.0	0.0
	20	0.1	0.1	0.1	0.1	0.0	0.0	0.0	0.0
7	1	0.5	1.0	0.7	0.6	0.2	0.4	0.2	0.2
	3	0.2	0.3	0.3	0.3	0.1	0.2	0.1	0.1
	5	0.2	0.2	0.2	0.2	0.1	0.1	0.1	0.1
	10	0.2	0.2	0.1	0.2	0.0	0.1	0.0	0.1
	20	0.2	0.2	0.1	0.2	0.0	0.1	0.0	0.0
10	1	0.6	1.3	0.9	0.8	0.2	0.5	0.3	0.2
	3	0.2	0.4	0.3	0.3	0.1	0.2	0.1	0.1
	5	0.2	0.3	0.2	0.2	0.1	0.1	0.1	0.1
	10	0.2	0.2	0.2	0.2	0.1	0.1	0.1	0.1
	20	0.2	0.2	0.2	0.2	0.1	0.1	0.1	0.1

**Table 5:** Minima on ZWD and height average standard deviations (STD) for each cut-off and two weighting functions, with the associated random walk. The rate of change (ROC) is calculated by comparing to the best average standard deviation presented on the first line of both ZWD and height parts.

Estimate	Cut-OFF	Weighting Function	Average STD	ROC	Associated Random Walk
	[°]		[mm]	[%]	[mm·h${}^{-1/2}$]
ZWD	3	sine	1.2		11
		sqrtsin	1.3	+5.3	5
	7	sine	1.4	+20.3	12
		sqrtsin	1.5	+24.9	7
	10	sine	1.7	+38.2	11
		sqrtsin	1.7	+39.5	7
height	3	sine	8.6		10
		sqrtsin	9.2	+6.6	8
	7	sine	8.9	+3.7	12
		sqrtsin	9.1	+6.3	8
	10	sine	9.2	+7.4	10
		sqrtsin	9.2	+7.1	10

**Table 6:** Average ZWD standard deviations obtained for sine and sqrtsin weighting functions for each of the underlined configurations of Table 5. The rate of change of the ZWD estimate obtained with sqrtsin compared to sine weighting function is given in the last column.

Estimate	Cut-Off	Random Walk	Average Standard Deviation		ROC
	[°]	[mm·h${}^{-1/2}$]	[mm]		
			**sine**	**sqrtsin**	
ZWD	3	11	1.2	1.5	+28.2%
		5	1.4	1.3	−9.8%
	7	12	1.4	1.7	+16.6%
		7	1.5	1.5	−1.9%
	10	11	1.7	1.8	+8.0%
		7	1.8	1.7	−4.8%

**Table 7:** ZWD and height average standard deviations for each cut-off and two weighting functions, for 5 mm·h${}^{-1/2}$ random walk. The rate of change is calculated by comparing sqrtsin to sine weighting function.

Estimate	Cut-Off [°]	Average Standard Deviation [mm]		ROC
		sqrtsin	sine	
ZWD	3	1.3	1.4	−9.8%
	7	1.5	1.7	−10.4%
	10	1.7	1.9	−11%
height	3	9.4	8.9	+5.9%
	7	9.4	9.2	+1.5%
	10	9.4	9.5	−1.5%

**Table 8:** Height and ZWD average standard deviation results for data sampling study.

Estimate	30 s (Ref.)	300 s	ROC
	[mm]	[mm]	
ZWD	1.3	1.8	+43.9%
Height	9.4	10.5	+11.9%
Correlation	0.45	0.62	+37%

**Table 9:** Height and ZWD average standard deviation results for GNSS constellation study.

Estimate	Multi-GNSS (Ref.)	GPS-Only	ROC
	[mm]	[mm]	
ZWD	1.3	1.9	+51.1%
Height	9.4	15.1	+59.8%
Correlation	0.45	0.40	−12.8%

**Table 10:** Average height and ZWD biases for different antenna movements over 24 h, processed with different antenna correction methods.

Vessel Movement	To North		45° North		24 h-Turn	
Average Bias [mm]	Height	ZWD	Height	ZWD	Height	ZWD
No correction	−8.5	−1.1	+6.2	+1.3	+9.0	+1.8
North correction	0	0	−1.8	−0.2	+1.0	+0.3
Azimuthal mean	−0.9	−0.2	+2.7	+0.4	+0.1	+0.1

**Table 11:** Height and ZWD average standard deviation increase rate compared to reference processing, for different antenna movements over 24 h, processed with different antenna correction methods in the selected configuration.

Vessel Movement	To North		45° North		24 h-Turn	
STD Increase	Height	ZWD	Height	ZWD	Height	ZWD
No correction	+7.6%	+10.6%	+5.4%	+11.7%	+9.9%	+22.0%
North correction	0	0	+4.1%	+10.0%	+6.1%	+16.6%
Azimuthal mean	+3.3%	+6.1%	+2.4%	+3.9%	+4.3%	+8.4%

**Table 12:** Height and ZWD average standard deviation results for study at low latitude (10°).

Estimate	Mid-Latitudes (Ref.)	Equator	ROC
	[mm]	[mm]	
ZWD	1.3	1.9	+48.0%
Height	9.4	12.2	+29.2%
Correlation	0.45	0.46	+1.7%

**Table 13:** Height and ZWD average standard deviation results for study at high latitudes (80°).

Estimate	Mid-Latitudes (Ref.)	Pole	ROC
	[mm]	[mm]	
ZWD	1.3	1.1	−10.9%
Height	9.4	10.3	+9.7%
Correlation	0.45	0.45	−0.1%

## Data Availability

Not applicable.

## Abbreviations

The following abbreviations are used in this manuscript:

GLONASS	GLObalnaïa NAvigatsionnaïa Spoutnikovaïa Sistéma
GNSS	Global Navigation Satellite System
GPS	Global Positioning System
IF	Ionosphere-free
JPL	Jet Propulsion Laboratory
MSL	Mean Sea Level
NGS	National Geodetic Survey
PCV	Phase Center Variation
PPP	Precise Point Positioning
RMSE	Root Mean Squared Error
ROC	Rate of Change
R/V	Research Vessel
STD	Standard Deviation
ZHD	Zenith Hydrostatic Delay
ZTD	Zenith Troposphere Delay
ZWD	Zenith Wet Delay
